# Small Angle Neutron Scattering Shows Nanoscale PMMA Distribution in Transparent Wood Biocomposites

**DOI:** 10.1021/acs.nanolett.0c05038

**Published:** 2021-03-18

**Authors:** Pan Chen, Yuanyuan Li, Yoshiharu Nishiyama, Sai Venkatesh Pingali, Hugh M. O’Neill, Qiu Zhang, Lars A. Berglund

**Affiliations:** † Beijing Engineering Research Centre of Cellulose and Its Derivatives, School of Materials Science and Engineering, Beijing Institute of Technology, 100081, Beijing, P.R. China; ‡ Department of Fibre and Polymer Technology, Wallenberg Wood Science Center, KTH Royal Institute of Technology, Teknikringen 56, 10044 Stockholm, Sweden; § Universite Grenoble Alpes, CNRS, CERMAV, 38000 Grenoble, France; ∥ Neutron Scattering Division and Center for Structural Molecular Biology, Oak Ridge National Laboratory, Oak Ridge, Tennessee 37831, United States

**Keywords:** Wood Nanotechnology, Neutron Scattering, Biocomposites

## Abstract

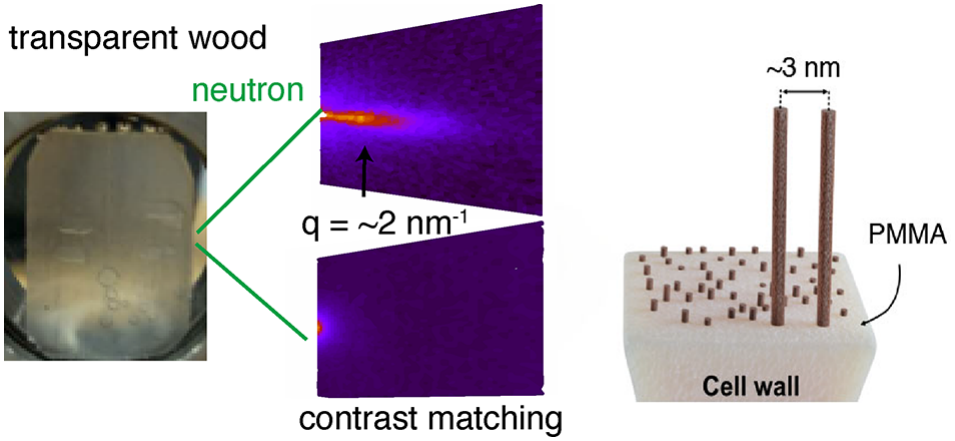

Transparent wood biocomposites based on PMMA combine high optical transmittance with excellent mechanical properties. One hypothesis is that despite poor miscibility the polymer is distributed at the nanoscale inside the cell wall. Small-angle neutron scattering (SANS) experiments are performed to test this hypothesis, using biocomposites based on deuterated PMMA and “contrast-matched” PMMA. The wood cell wall nanostructure soaked in heavy water is quantified in terms of the correlation distance *d* between the center of elementary cellulose fibrils. For wood/deuterated PMMA, this distance *d* is very similar as for wood/heavy water (correlation peaks at *q* ≈ 0.1 Å^–1^). The peak disappears when contrast-matched PMMA is used, indeed proving nanoscale polymer distribution in the cell wall. The specific processing method used for transparent wood explains the nanocomposite nature of the wood cell wall and can serve as a nanotechnology for cell wall impregnation of polymers in large wood biocomposite structures.

Strong polymer matrix nanocomposites are of interest to extend the property range of composites, and with nanostructural control such nanocomposites can combine functional and structural (load-bearing) properties. For instance, researchers at Toyota demonstrated improved mechanical properties by dispersing strong clay platelets in a thermoplastic matrix in which optical transmittance, gas barrier properties, and thermal stability were also improved.^1^ Biocomposites produced based on fibrous cellulose facilitate sustainable development of our society by the utilization of renewable resources, and the potential for lower carbon foot print and lowered processing energy. Fibrous cellulose is synthesized by plants absorbing CO_2_, whereas petroleum-based polymer fibers require multiple chemical processing steps with substantial energy demands.

Cellulose nanocomposites with a polymer matrix can combine excellent mechanical properties with low thermal expansion, high optical transmittance,^2^ and low moisture sorption.^3^ It is also straightforward to add functional properties, for example, for photonics applications.^4^ In addition, many nanocellulose processing methods are scalable,^5^ paving the way for large-scale, eco-friendly nanotechnologies. However, bottom-up approaches for nanocomposites preparation suffer two major challenges. First, there is a need for substantial processing energy to first disintegrate nanofibrils from plant tissue but also to assemble the nanocomposite. Second, it is difficult to achieve high orientation and good dispersion of fibrous nanoparticles in polymer matrices, and poor nanoparticle dispersion leads to severely compromised physical properties.^6^

Because of the challenges mentioned, top-down preparation of cellulose nanocomposites based on preserved wood nanostructure is very attractive. In wood, the so-called “elementary” cellulose fibrils of about 3 nm in diameter are already preferentially oriented in the axial wood fiber direction and well dispersed at nanoscale. Large-scale structures can be prepared, and recently wood has been modified to produce functional materials and devices^7^ relying on state-of-the-art nanotechnology.^8^

Thermoplastic biocomposites have been prepared from wood substrates impregnated by styrene or methyl methacrylate (MMA) monomers and polymerized into wood/polystyrene and wood/PMMA biocomposites to reduce swelling or shrinkage from humidity changes.^9^ If the MMA monomer is impregnated in liquid bulk form, the polymer matrix only fills the empty lumen space of wood^9^ (Figure 1). Under these conditions, the monomers are unable to diffuse into the wood cell wall. In recent development of multifunctional wood–polymer composites, lignin chromophores are removed from the wood substrate to form wood–poly(methyl methacrylate) (PMMA) composites,^10^ termed “transparent wood” due to high optical transmittance. Transparent wood has been functionalized into a variety of photonic materials and devices, including luminescent nanocomposites from quantum dots and a wood laser.^11^

**Figure 1:**
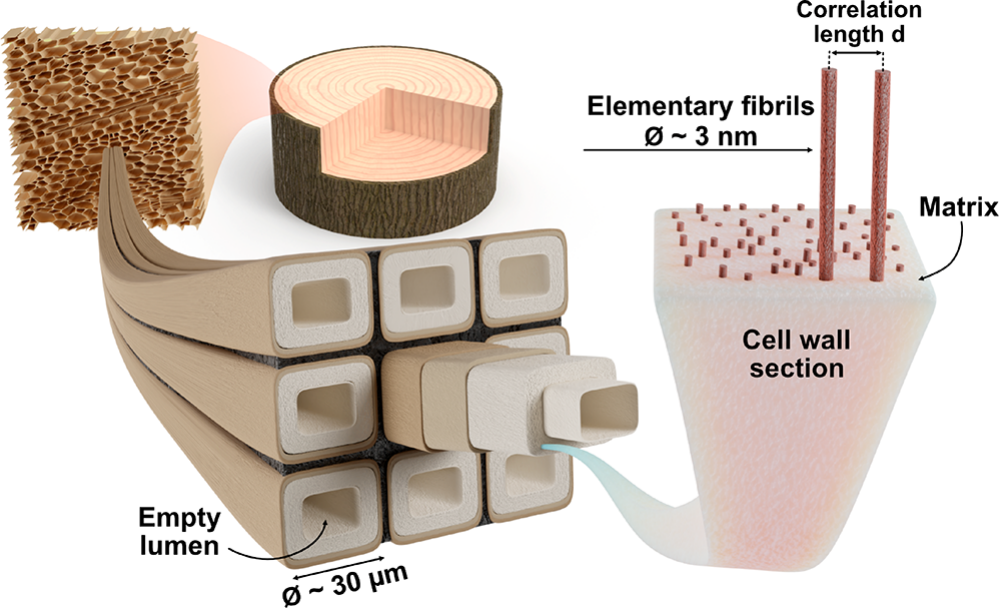
Cellular structure in wood, showing nine wood fibers and an enlarged view of a section of the cell wall nanostructure. Elementary cellulose fibrils are distributed in a “matrix”, and the interfibril correlation length *d* between fibril centers is estimated using small-angle neutron and X-ray scattering (SANS/SAXS). Different samples have different compositions of the “matrix”, see Table 1.

Native wood (NatW) in a living tree is structured over multiple scales (Figure 1). Wood can be viewed as an assembly of tubular fiber cells with empty lumen space in the center. The cell wall in turn contains cellulose fibrils at nanoscale. At this scale in NatW, strong elementary cellulose fibrils (∼3 nm diameter) are preferentially oriented in the axial fiber direction and embedded in an amorphous “matrix” of hydrated hemicelluloses and lignin.^12−14^ During delignification, lignin and part of the hemicelluloses are removed. The nanostructure of the hydrated delignified wood (DelW) is unknown, but the “matrix” composition in Figure 1 is water and amorphous hemicelluloses, which potentially functions as a “spacer” that hinders agglomeration of neighboring cellulose microfibrils.^15,16^ The removal of lignin, followed by impregnation of a polymer with similar refractive index as cellulose, results in optically transparent composites with improved mechanical performance.^10^ Although physical properties indicate that PMMA is present in the cell wall, this has not been confirmed and the polymer distribution is unknown.

The presence of a polymer in the lumen space of the composite can be verified by microscopy techniques.^10,17^ For some polymers, their location inside the cell wall can be verified by optical Raman microscopy,^18^ but the resolution is limited and data interpretation is not straightforward for PMMA mixed with wood biopolymers. Transmission electron microscopy (TEM) of ultramicrotomed cross sections is also potentially useful for nanoscale cell wall structures, but the harsh microtome cutting process as well as staining techniques will alter nanoscale structure.

During transparent wood preparation, swollen DelW is infiltrated by a MMA monomer solution. Possibly, the MMA monomers not only infiltrate the microsized lumen space but also the wood cell wall matrix. This is simpler than grafting from polymerization inside the cell wall^19^ and technically interesting. The use of deuterated MMA (D-MMA) combined with neutron scattering should make it possible to investigate if the present transparent wood is a true polymer matrix nanocomposite in that the polymer is distributed at nanoscale in the wood cell wall “matrix”,^20^ see Figure 1.

In the present study, both SANS and SAXS were used to investigate the nanoscale cell wall structure. SAXS provides nanostructural information based on the contrast in electron density between different components. D_2_O swollen wood shows characteristic SANS peaks obtained from D_2_O/cellulose fibril contrast. The peaks are due to elementary cellulose fibril–fibril correlation.^21,22^ The nanoscale PMMA and elementary cellulose fibril distribution is successfully investigated in modified wood cell walls, and the results clarify nanoscale processing mechanisms. The methodologies can provide nanostructural insight of thermoplastic nanocomposites from wood substrates, which is critical in order to tailor mechanical and functional properties.

The preparation process for transparent wood based on PMMA can be summarized in four steps (Scheme 1). (1) From NatW (1 mm × 20 mm × 20 mm plane cut birch wood), the light-absorbing lignin component is removed to form DelW,^25^ (2) solvent exchange of the white DelW substrate from water to less polar solvents, (3) infiltration of MMA monomer into DelW assisted by solvent evaporation, and (4) thermal polymerization. Seven wood materials were prepared through the procedure described in Scheme 1: NatW, DelW, dried DelW, a transparent wood biocomposite with fully deuterated PMMA matrix (DelW/D-PMMA), with fully hydrogenated PMMA matrix (DelW/PMMA), with a mixture of deuterated and hydrogenated MMA matrix (DelW/partD-MMA), and with partially deuterated PMMA matrix (DelW/partD-PMMA). The materials investigated by SANS are listed in Table 1.

**Scheme 1:**
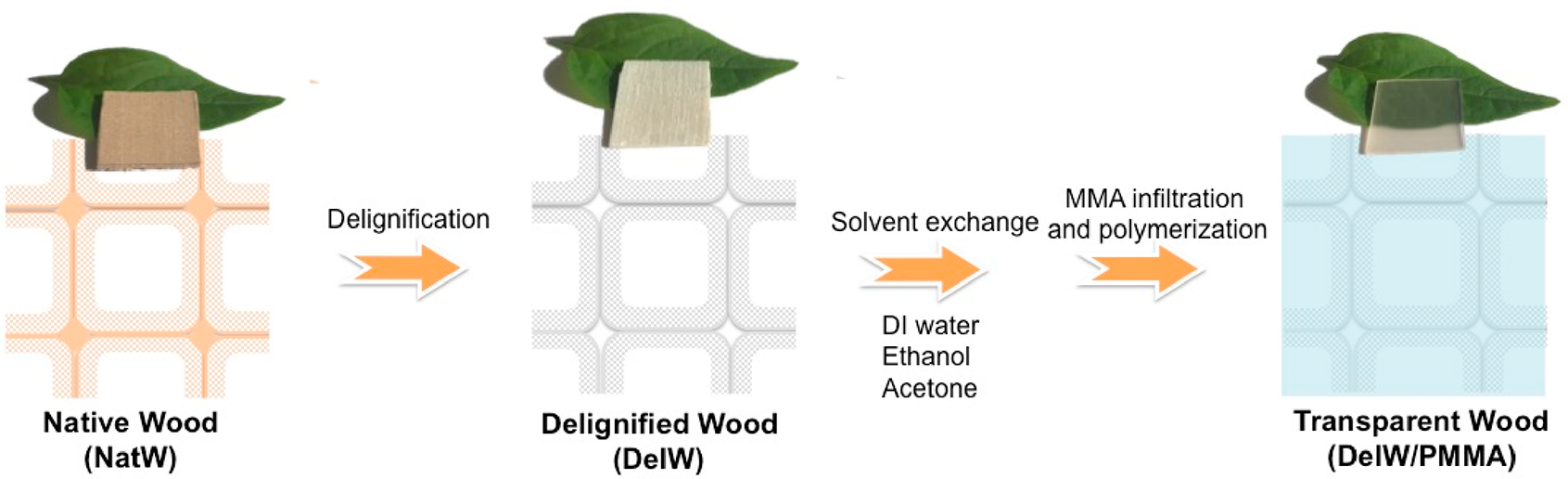
Preparation Procedure from NatW to DelW/PMMA NatW was Processsed by Delignication, Solvent Exchange, MMA Monomer Infiltration Followed by Polymerization to Form Transparent Wood in the Form of DelW/PMMA.

**Table 1:** Material Designations and Descriptions

designation	description
NatW/D_2_O	native wood soaked in D_2_O. “Matrix” in Figure 1 is a molecular mixture of D_2_O, hemicellulose, and lignin
DelW/D_2_O	delignified wood soaked in D_2_O; “Matrix” is D_2_O and hemicelluloses
dried DelW	freeze-dried delignified wood
DelW/D-PMMA	DelW impregnated by deuterated D-MMA, polymerized into D-PMMA to form transparent wood
DelW/PMMA	same as above, but based on conventional hydrogenated MMA
DelW/partD-MMA	DelW impregnated by a 78–22% mixture of MMA and D-MMA, to remove scattering contrast of MMA region
DelW/partD-PMMA	same as above, but after polymerization

Transparent wood prepared from birch according to Scheme 1 is a multifunctional material with an optical transmittance of 81% at 1 mm thickness (wavelength of 550 nm), a Young’s modulus of 19 GPa, and an ultimate tensile strength of 270 MPa.^26^ This corresponds to an effective cell wall modulus of 70 GPa, and a cell wall strength of 960 MPa based on simple micromechanics for composites.^24^ The possible distribution of PMMA inside the cell wall of this cellulosic biocomposite could partly explain these exceptional properties.

An essential aspect of the preparation (Scheme 1) is that the wood substrate was kept in swollen, “never-dried” state, to avoid nanostructural changes in the form of cellulose fibril agglomeration^24^ from drying. Neutron scattering of NatW and DelW soaked in deuterated water (D_2_O) give information on the correlation length *d*, the center-to-center distance between the cellulose fibrils (see Figure 1) before and after delignification. For the final biocomposite, transparent wood in the form of DelW/D-PMMA is the main material for SANS characterization of nanostructure.

The two-dimensional SANS patterns of all materials in Table 1 are presented in Figure 2 with corresponding anisotropic and isotropic scattering profiles in Figure 3.^23^ The materials are collected at different stages of the preparation process in order to learn about nanoscale mechanisms. The fitting procedures for the SANS curves are presented in SI (Figure S1 and S2). Strong anisotropic streaks in the direction perpendicular to the longitudinal direction (equatorial) were observed for all materials (Figure 2A–E), except for the two samples where the DelW substrate was impregnated by a mixture of deuterated monomer (D-MMA) and hydrogenated MMA, and then polymerized into partD-PMMA in which case the scattering was dominantly isotropic (Figure 2F). In a qualitative sense, the dried DelW material (Figure 2C) is different from the D_2_O-soaked wood materials (Figure 2A,B) in that the wing detectors do not show a distinct streak.

**Figure 2:**
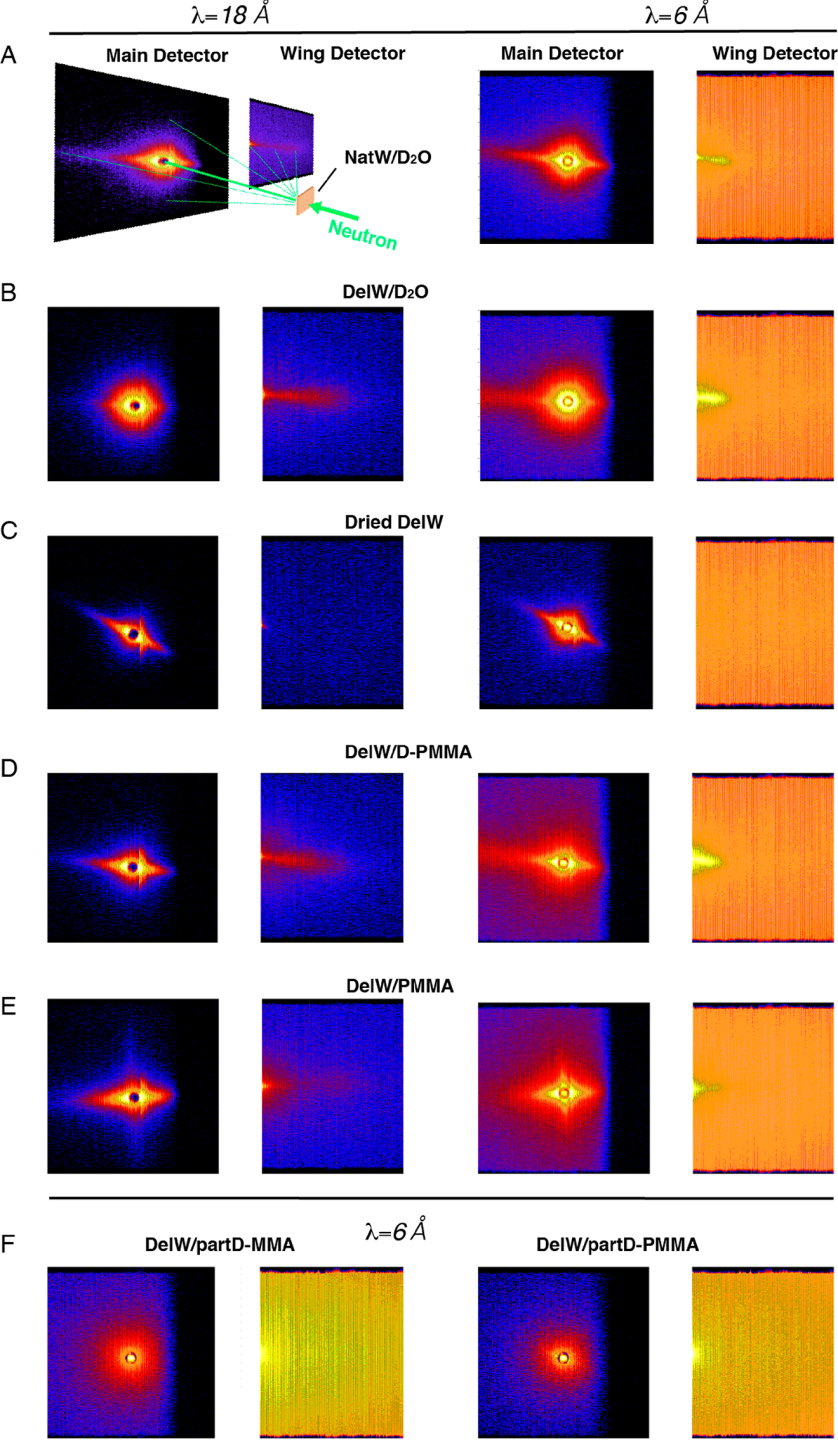
Two-dimensional SANS patterns at two incident neutron wavelengths. (A) NatW/D_2_O; (B) DelW/D_2_O; (C) dry DelW; (D) DelW/D-PMMA; (E) DelW/PMMA; (F) DelW/partD-MMA and DelW/partD-PMMA. Material designations are explained in Table 1.

**Figure 3:**
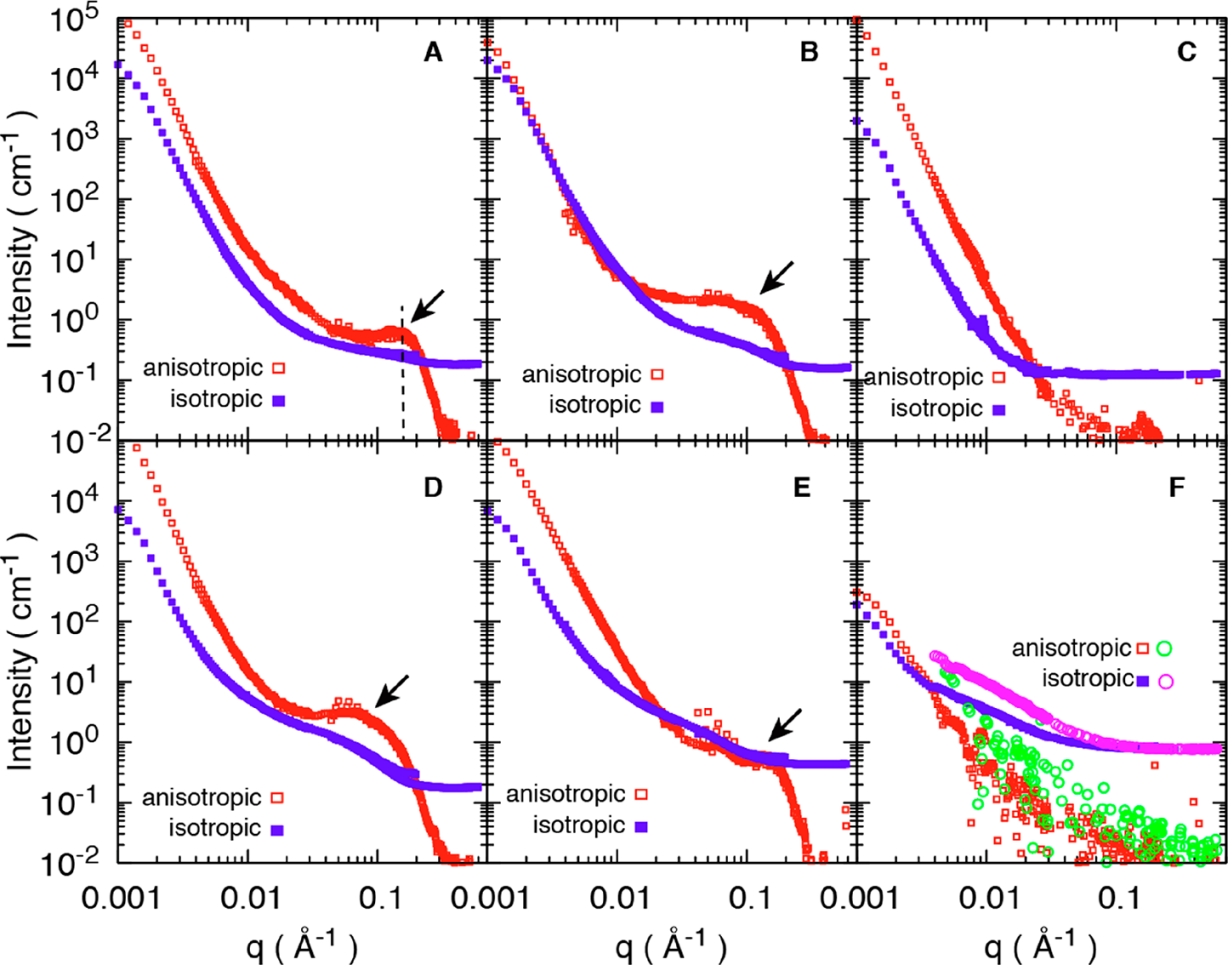
Decomposed anisotropic and isotropic SANS profiles from materials listed in Table 1: NatW/D_2_O (A), DelW/D_2_O (B), dry DelW (C), DelW/D-PMMA (D), DelW/PMMA (E), and DelW in liquid partD-MMA mixture (F, squares), and DelW/partD-PMMA (F, circles). Note that two materials are presented in (F). Arrows in the figures indicate the shoulder or peaks present in the high *q* region.

The data shown in Figure 2 are raw data as captured on the detector. The azimuthal distribution depends on the *q*-range as it reflects the structural features at different length scales, and each scattering intensity is the result of structure and the contrast, which requires further treatment for interpretation. However, even without any data reduction, the comparison of Figure 2D,F directly shows that the isotopic change of PMMA leads to a large effect on scattering, and that materials in Figure 2B,D are similar, as will be discussed in detail.

The structure of NatW soaked in D_2_O is an important reference material, see Figures 2A and 3A. Apart from water in the lumen space, the cell wall contains about 30 wt % of D_2_O in the soaked condition.^22,27^ The ∼20 μm diameter lumen pore space, see Figure 1, is filled with D_2_O so that the neutron scattering density becomes significantly different from the cell wall. The straight line with a downward slope (left part of Figure 3A) in the low *q* region (0.001–0.004 Å^–1^) arises from the surface of the larger scale lumen structure, that is, the interface between D_2_O in lumen space and the D_2_O-swollen cell wall, see Figure 1. The intensity in Figure 3A scales with *q*^–4^, which is characteristic for flat surfaces. In Figure 3A (arrow), there is an intensity peak at *q* = 0.16 Å^–1^ (from fitting, Figure S1), which is interpreted using Bragg’s law^14^,^21^*d* = 2π/*q*, where *d* is the interfibril correlation length (Figure 1), so that *d* ≈ 3.9 nm. This reflects the correlation in the arrangement of oriented elementary cellulose fibrils in the wood cell wall. Note that the distribution of fibrils in the wood cell wall is less ordered than in crystals, where *d* corresponds to the interplanar spacing. If we assume that the elementary fibril is 3 nm in diameter, the distance between two neighboring fibril surfaces (matrix region) would then be ∼0.9 nm for native wood soaked in D_2_O. On the basis of Figure 1, this region in native wood is a D_2_O-swollen “matrix”, which also contains hemicelluloses and lignin.^28^ The peak is a result of the contrast between the crystalline core of cellulose microfibrils (scattering length density SLD 1.87 × 10^–6^ Å^–2^) and the surrounding hemicellulose-lignin mixture swollen in D_2_O (SLD 6.39 × 10^–6^ Å^–2^). This interpretation has been confirmed for wood, since the peak disappears when D_2_O and H_2_O are mixed at a D/H ratio of 0.35:0.65,^13^ so that the neutron scattering densities in cellulose and the “matrix” become equal.

The isotropic scattering intensity in Figure 3A in the low *q* region is mainly surface scattering and is 1 order of magnitude lower than the anisotropic scattering arising from the shape of the tubular libriform fiber cells in birch wood. They are 14–40 μm in diameter and 1.1–1.2 mm in length with a cell wall thickness of 3–4 μm.^29^

Figure 3B is for DelW kept in never-dried state and solvent exchanged to D_2_O. Compared with lignin-containing NatW/D_2_O, this did not change the general scattering features of the anisotropic component (Figure 3B, Figure S1), although a shift to a lower angle (smaller *q*, 0.11 Å^–1^) was observed which corresponds to a larger interfibrillar correlation length (*d* ∼ 5.7 nm). If the elementary cellulose fibril has a diameter of 3 nm, the distance between neighboring fibril surfaces for DelW/D_2_O would be ∼2.7 nm. This increased spacing compared with NatW/D_2_O is due to the cell wall swelling after lignin removal from the cell wall. When DelW was dried, the scattering features at high *q* were completely lost, see Figure 3C. Such featureless scattering could be ascribed to the reduced contrast between matrix and microfibrils due to the replacement of D_2_O with air as well as structural change induced by drying.

It is interesting that the anisotropic scattering does not change drastically by delignification treatment (Figure 3B), which means that the treatment is mild and the nanostructure is well preserved. This is in contrast to other reports where pulping or hydrothermal pretreatment leads to more drastic structural changes.^24^ The present process is specific for lignin removal at relatively low treatment temperature, thus preventing extensive leaching of hemicelluloses, which will otherwise lead to agglomeration of neighboring elementary cellulose fibrils.^30^

The material in Figure 3D is the deuterated transparent wood composite; a delignified wood substrate with D-PMMA filling the pore space (DelW/D-PMMA). The observation of a peak at 0.09 Å^–1^ in the anisotropic scattering, see Figure 3D and Figure S1, is very important. It confirms that the spatial arrangement of cellulose microfibrils and the D-PMMA “matrix” phase (with some hemicelluloses) is such that the polymer is distributed at the nanoscale between individual microfibrils. The interfibril correlation length *d* of DelW/D-PMMA (7.0 nm) is even slightly larger than for the swollen DelW/D_2_O material (5.7 nm), and the processing protocol results in the formation of a true DelW/D-PMMA nanocomposite in the wood cell wall. One possible explanation is partial agglomerates of fibrils in the cell wall, resulting in average, relatively larger interfibrillar correlation length *d*, reflecting the presence of a fraction of larger “fibrils”. The scattering contrast between D-PMMA and the DelW substrate is high and presented in Table 2. If conventional MMA is used for impregnation and polymerization to form DelW/PMMA, the scattering features are much less apparent (Figure 3E). The confirmed nanoscale distribution of PMMA is significant: polymer molecules will have different conformations (and properties) in confined space, compared with bulk state.^31^

**Table 2:** Coherent Neutron Scattering Length (NSL) and Scattering Length Density (SLD)

	hydrogenated			deuterated		
	density (g/mL)	NSL (× 10^–12^ cm)	SLD (× 10^–6^ Å^–2^)	density (g/mL)	NSL (× 10^–12^ cm)	SLD (× 10^–6^ Å^–2^)
water	1.0	–0.168	–0.56	1.11	1.92	6.39
MMA	0.94	1.492	0.84	1.011	9.82	5.53
PMMA	1.18	1.492	1.06	1.274	9.83	6.97
cellulose	1.60	3.150	1.87			

As a final verification of the proposed nanocomposite structure in Figure 1D with PMMA and hemicellulose as the matrix, the neutron scattering length density of the MMA matrix was tailored to match that of cellulose in order to render no scattering contrast between cellulose and the nanoscale matrix phase. The DelW substrate was impregnated by a mixture of deuterated D-MMA and conventional MMA (22:78) termed partD-MMA. The scattering contrast is effectively null since this partD-MMA mixture has a neutron scattering length density similar to the DelW wood component. Indeed, we observe no anisotropic scattering as observed in Figure 2F and its decomposed 1D scattering profiles in Figure 3F. The anisotropic scattering is now very much weaker than the isotropic counterpart. It confirms that the peak in deuterated transparent wood (DelW/D-PMMA) is due to the contrast between DelW and the D-PMMA, since this is the only parameter modified. The contrast matching point of D-PMMA to cellulose is 13.8% (Figure 4A), slightly lower than that of D-MMA (22.0%), therefore there is a slight contrast difference when the monomer MMA is polymerized into PMMA polymer. This is confirmed by the slightly larger anisotropic signal in DelW/partD-PMMA compared with DelW/partD-MMA (Figure 3F, green circle versus red square).

**Figure 4:**
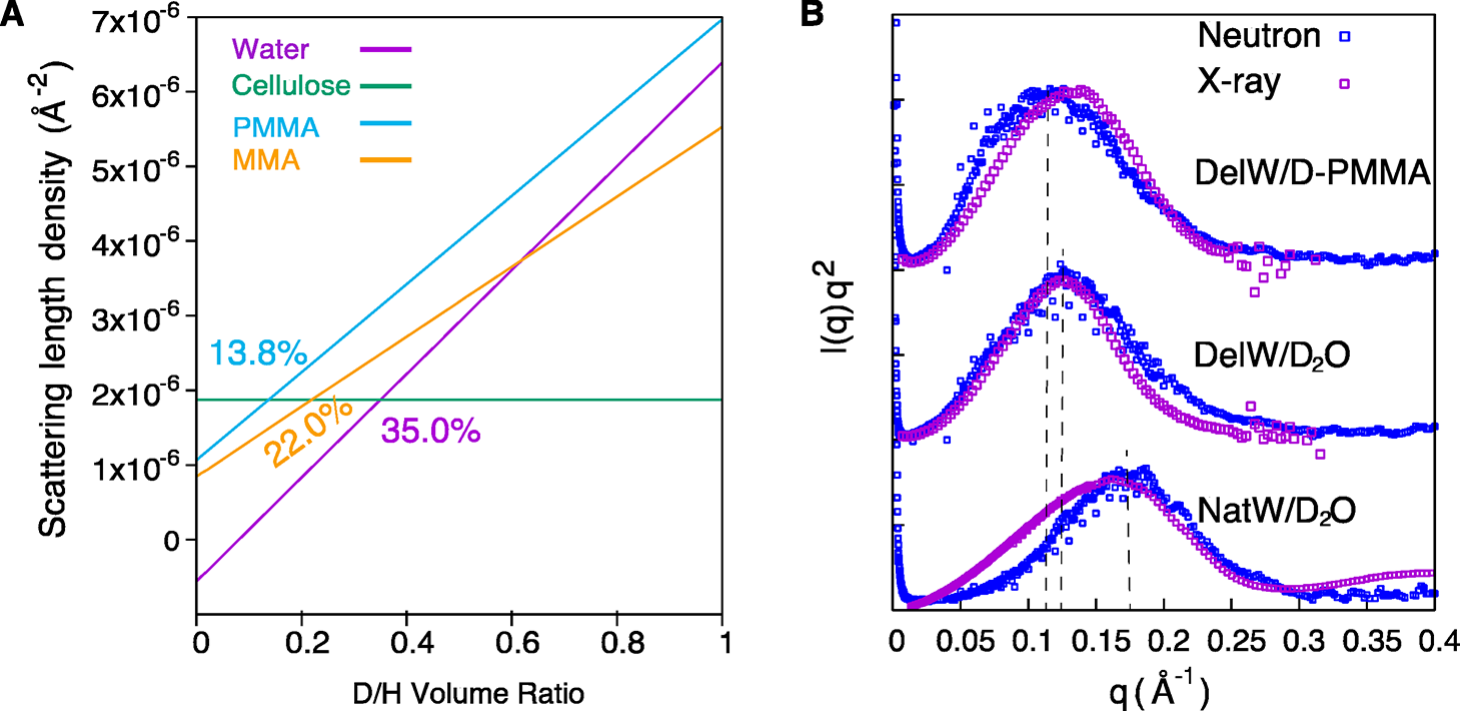
Neutron scattering length density of cellulose, water, PMMA, and MMA as a function of deuterium/hydrogen D/H ratio (A). The *I*(*q*)*q*^2^ profiles from SANS and SAXS for NatW/D_2_O, DelW/D_2_O, and DelW/D-PMMA (B).

Our key result is the nanoscale distribution of PMMA in the transparent wood biocomposite; PMMA is not just distributed inside the cell wall but is also located in the interfibril regions that are a few nanometers in dimension. Since PMMA is known to be incompatible with hydrophilic polysaccharides such as cellulose and hemicelluloses, the nanoscale PMMA distribution is an achievement of immense value. The main reason is the solvent-assisted process of MMA monomer impregnation. The process finds some parallel to the native lignification process *in vivo*, where monolignol glucoside precursors impregnate the cell wall, then polymerize into lignin after cleaving the water-soluble glucose moiety.^32^ Here, the water-swollen cell wall was subjected to solvent exchange and MMA diffused into the cell wall, followed by polymerization into PMMA. MMA is slightly more polar than its polymerized form due to the ester groups. The mild delignification left the DelW cell wall in a state very similar to that before lignification, and hemicellulose functions as a spacer between elementary cellulose fibrils impeding their coalescence.^33^

For further support, SAXS profiles are presented in Figure S3 and confirm the trends in corresponding SANS profiles. Electron densities for cellulose, hemicellulose, lignin, PMMA, and water are presented in Table S1, and the contrast between cellulose and water-swollen hemicellulose results in the scattering peaks around 0.1 A^–1^ for NatW and DelW soaked in water. For DelW/PMMA, the contrast is mainly between cellulose and PMMA. In the WAXS data, see Figure S4, the interpretation of elementary cellulose fibrils is confirmed from the decomposed anisotropic curves with typical diffraction profiles of crystalline cellulose (11̅0, 110 and 200) in all three materials (NatW/H_2_O, DelW/H_2_O, and DelW/PMMA), where the isotropic curve mainly corresponds to PMMA. The main advantage of SANS is the adjustment of the “matrix” contrast in the DelW/partD-MMA and DelW/partD-PMMA samples, confirming the origin of the SANS peak. Detailed information about semiquantitative interpretation of X-ray/neutron scattering peak position on woody samples is shown in Supporting Information, Part 2.

Figure 4B shows the I(*q*)*q*^2^ plot from SANS and SAXS for NatW, DelW, and DelW/D-PMMA materials. SAXS data are in broad agreement with SANS data and the materials have peak positions at 0.17, 0.125, and 0.11 Å^–1^, corresponding to interfibril correlation lengths *d* of 3.7 nm for native wood NatW/D_2_O, 5.0 nm for delignified wood DelW/D_2_O and 5.7 nm for DelW/D-PMMA respectively. There were slight differences in peak positions between SANS and SAXS, (Figure 4B). Note that the peak is the convolution of the unknown form-factor of elementary cellulose fibrils and the correlation between center-to-center distance of the fibrils, which means that the distances should not be interpreted as exact (Cf. Supporting Information Part 2). The neutron scattering density is sensitive to isotope exchange between hydrogen and deuterium. Since the surface of elementary cellulose fibrils is deuterated in aqueous media, the effective diameter of elementary cellulose fibrils as scattering objects becomes somewhat reduced, and the form factor extends to a higher angle.^34^ For MMA, this isotope exchange does not occur since MMA does not deprotonate, and thus the peak position for the DelW/PMMA materials is closer to X-ray data.

In conclusion, the presence of a peak in the SANS profiles of our transparent wood-PMMA biocomposites reveals the nanoscale presence of PMMA in interstices of ∼3 nm wide cellulose microfibrils inside the cell wall, confirming its nanocomposite structure. This is an important reason why this transparent wood material, based on birch, shows high optical transmittance and excellent mechanical properties. Rayleigh-scattering from small scale optical defects is reduced, and stress transfer is improved by the nanoscale distribution of polymer inside the cell wall.

Wood samples were not allowed to dry during processing which preserved the spatial arrangement of elementary cellulose fibrils throughout the processing stages with some limited cell wall swelling observed as lignin was removed. Assisted by the solvent-exchange procedure, MMA is able to diffuse into the cell wall and polymerize. The average distance between neighboring fibril surfaces, filled by a PMMA (and hemicellulose) “matrix”, was estimated to be at the scale of ∼3 nm. The monomer impregnation method may be a scalable nanotechnology, since a related processing concept is used in industrial production of microstructured carbon fiber/epoxy prepreg. Alternatively, scalable production of bulk transparent wood is feasible through plywood technology.

## Supporting Information Available

Part 1: details of materials and methods; (Table S1) electron density scattering length (EDSL) of wood components, MMA, PMMA, and water; (Table S2) fitted parameters of anisotropic and isotropic SANS profiles; (Figure S1) fitting of anisotropic SANS profiles; (Figure S2) fitting of the isotropic SANS profiles; (Figure S3) superposition of anisotropic SAXS and SANS curves; (Figure S4) decomposed WAXS profiles of NatW/H_2_O, DelW/H_2_O, DelW/PMMA, and PMMA. Part 2: Semiquantitative interpretation of X-ray/neutron scattering peak position on woody samples; (Figure S5) schematic illustration of normalized form factor, pair distribution function, and structure factor of densely packed cylinders; (Figure S6) effect of cylinder form factor on the peak position; explanation on the origin of the differences in peak position between X-ray and neutron scattering (PDF)
