# Supplementary material for: Small Angle Neutron Scattering Shows Nanoscale PMMA Distribution in Transparent Wood Biocomposites
Source: Nano Lett. 2021 Mar 18;21(7):2883–90. doi: 10.1021/acs.nanolett.0c05038 (PMC8050824; doi:10.1021/acs.nanolett.0c05038)
Supplement: Supplementary file 1 — nl0c05038_si_001.pdf [file nl0c05038_si_001.pdf]

Supporting information of "Small angle neutron scattering shows nanoscale PMMA distribution in transparent wood biocomposites"

Pan Chen[1,*,†], Yuanyuan Li[2,†], Yoshiharu Nishiyama[3,*], Sai Venkatesh Pingali[4], Hugh M. O'Neill[4], Qiu Zhang[4], Lars A Berglund[2,*]

[1]Beijing Engineering Research Centre of Cellulose and Its Derivatives, School of Materials Science and Engineering, Beijing Institute of Technology, 100081, Beijing, P.R. China

[2]Department of Fibre and Polymer Technology, Wallenberg Wood Science Center, KTH Royal Institute of Technology, Teknikringen 56, 10044 Stockholm, Sweden

[3]Universite Grenoble Alpes, CNRS, CMERAV, 38000 Grenoble, France

[4]Center for Structural Molecular Biology, Oak Ridge National Laboratory, Oak Ridge, TN 37831, USA

**Corresponding author: panchen@bit.edu.cn , yoshi@cermav.cnrs.fr , blund@kth.se ;*

† PC and YL contribute equally.

Part 1.

Materials and Methods:

Small angle neutron scattering (SANS):

SANS measurements were carried out using CG-3 Bio-SANS instrument at the High Flux Isotope Reactor (HFIR) facility in Oak Ridge National Laboratory (ORNL).[1] The instrument is equipped with a wing detector and a central main detector with a distance to sample of ~1 m and ~15 m, respectively. Two neutron wavelengths (λ) of 6 Å and 18 Å were used to cover a wide q range from 0.001 to 1 Å^{-1}.

The neutron scattering intensities were normalized to incident beam intensity and corrected for detector dark current, pixel sensitivity and scattering from the quartz windows of the titanium cells. The anisotropic and isotropic scattering intensity profiles were decomposed by fitting the azimuthal intensity distribution at each q with two Gaussian peaks and a constant as described by Nishiyama et al.[2]

The samples were placed in a titanium sample holder, either being filled with air or with D_2O. The NatW and DelW samples were immersed in heavy water under vacuum to ensure trapped air in pores were also replaced with heavy water and left sealed for 24 hours. Dried DelW substrates were prepared by freeze-drying before D_2O infiltration. Each sample was collected for 2 hours at each incident wavelength.

Small/Wide angle X-ray Scattering (SAXS/WAXS):

The small/wide angle X-ray scattering experiment was performed at D2AM beamline in European Synchrotron Radiation Facility (ESRF, Grenoble, France) with the X-ray energy of 18 keV and at LiX ID-16 of NSLS-II in Brookhaven National Laboratory (BNL). At D2AM, two silicon pixel detectors, WOS and D5 (ImXPad, currently succeeded by Rubirx) were used simultaneously to collect wide and small angle scattering (19 cm and 220 cm). The gap region was further measured by placing D5 detector at 39 cm without WOS detector. The anisotropic and isotropic scattering profiles were extracted from the two dimensional scattering patterns using a numerical approach as described by Nishiyama et al.[2]

Sample preparation:

One-millimeter-thick plane-cut birch wood veneer was purchased from Glimakra of Sweden AB. Pieces of 1 mm x 20 mm x 20 mm were cut from the veneer and used as starting materials.

The DelW is prepared through a delignification process, which consists of immersing the native wood chips in aqueous $NaClO_2$ solution under pH of 4.6 for 6 hours until the color turns white. The resulting wood structure has low lignin content (1.5%) and is a suitable template for various modification approaches of the nanostructure.[3] The DelW was solvent-exchanged without drying in the sequence of water, ethanol, and acetone, three times for each solvent. The acetone-containing DelW substrate was then directly immersed in pre-polymerized MMA solution at room temperature. Under vacuum, the acetone was evaporated from the substrate, as MMA replaced the acetone. The MMA impregnated DelW substrates were sandwiched between two glass plates and sealed with aluminum foil followed by placing in an oven at 70 °C for 4~6

hours for MMA polymerization. Three different transparent wood samples were prepared with different isotopic composition of MMA: Conventional methyl methacrylate (MMA, Sigma-Aldrich), Deuterated methyl methacrylate (D-MMA, Sigma-Aldrich), and partD-MMA (78% wt. MMA + 22% wt. D-MMA). More detailed protocol was described previously.[4]

Table S1. Electron density scattering length (EDSL) of wood components, MMA, PMMA, and water, calculated through online Neutron/X-ray scattering Length Density Calculator (https://sld-calculator.appspot.com), assuming a chemical formula of $C_6H_{10}O_5$ for cellulose, $C_5H_8O_4$ for hemicellulose, and $C_{81}H_{92}O_{28}$ for Organosolv lignin. The density of cellulose is considered as 1.60 g/mL[5,6], and that of hemicellulose and lignin is taken as 1.52 g/mL and 1.40 g/mL, respectively, reported by Ehrnrooth[7].

	Density ($g\ mL^{-1}$)	EDSL
H_2O	1.0	9.45
MMA	0.94	8.64
PMMA	1.18	10.84
Cellulose	1.60	14.47
Hemicellulose	1.52	13.73
Lignin	1.40	12.26

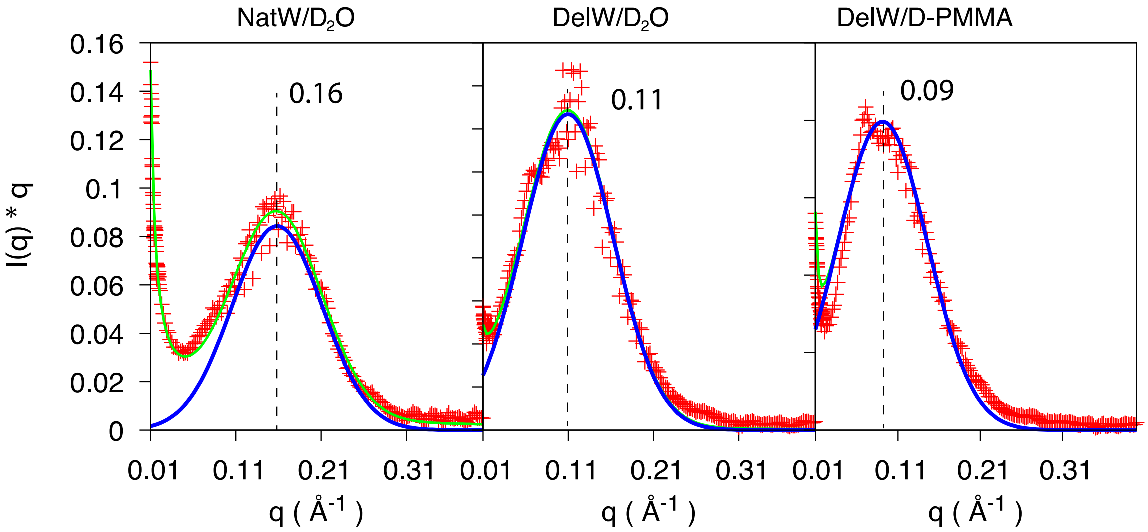

Figure S1: Fitting of anisotropic SANS profiles (I(q)×q) at relative high *q* range (0.01-0.5 Å^{-1}) using two decay power law (-3 & -1) and one gaussian function for various samples. The fitted parameters are summarized in Table S2

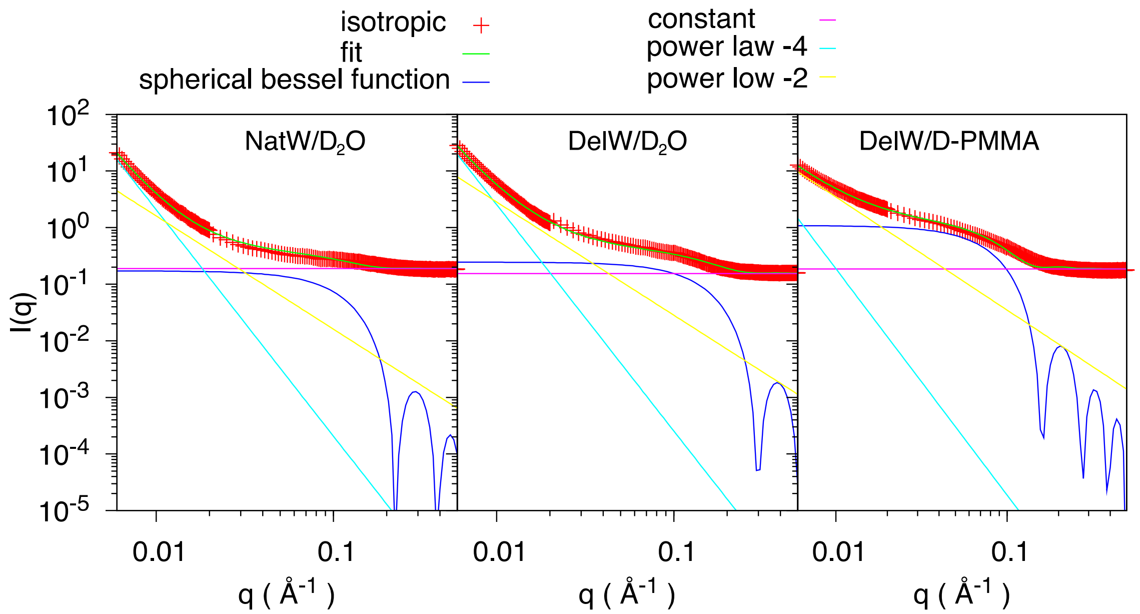

Figure S2: Fitting of the isotropic SANS profiles using two decay power law (-4 & -2) and one spherical Bessel function for various samples. The fitted radiuses of gyration are 2, 1.5 and 2.8 from left to right.

The anisotropic scattering profiles were fitted using two decay power law (-3 & -1) and one gaussian function as described:

$$I(q) = a \exp(-((q - b)/c)^2) + d\, q^{-3} + g\, q^{-1} \quad (1)$$

This isotropic scattering profiles were fitted using two decay power law (-4 & -2) and one spherical Bessel function and a constant as described:

$$I(q) = \frac{(\sin(aq) - aq\cos(aq))}{(aq)^3} c + d + fq^{-4} + bq^{-2} \quad (2)$$

Table S2: The fitted parameters of anisotropic and isotropic SANS profiles in equation (1) and (2).

Anisotropic					
	a	*b* (Å^{-1})	*c*	*d*	*g*
NatW/D2O	0.0843064	0.158463	0.0749381	0.00098	4.92 × 10^{-8}
DelW/D2O	0.146848	0.109774	0.0754	0.0002	3.6 × 10^{-9}
DelW/PMMA	0.199143	0.0915442	0.0770501	3.48× 10^{-8}	7.6 × 10^{-8}
Isotropic					
	a (Å)	*b*	*c*	*d*	*f*

NatW/D2O	19.9602	1.60646	1.54619	0.188905	2.06707
DelW/D2O	15.0365	2.8333	2.21508	0.154853	2.54769
DelW/PMMA	28.0479	3.49256	9.77555	0.185265	0.188576

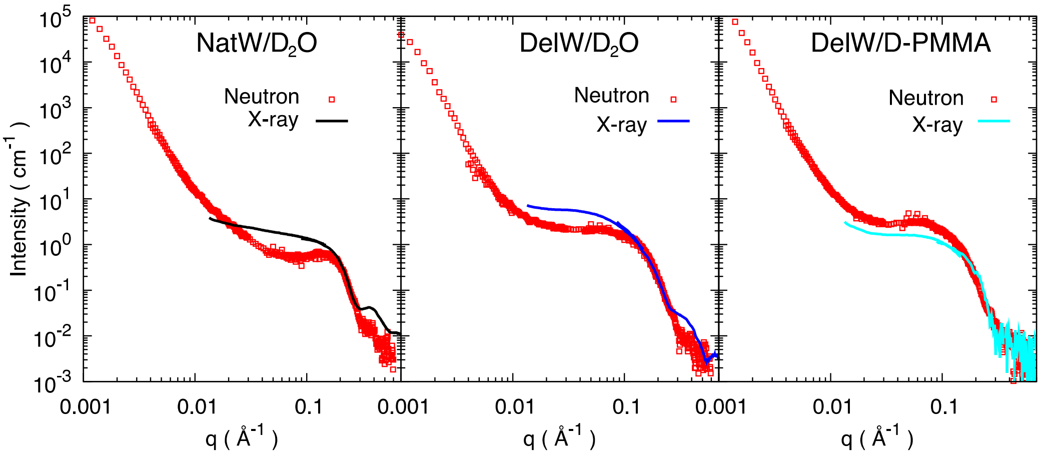

Figure S3: The superposition of anisotropic SAXS and SANS curves for NatW/D_2O, DelW/D_2O and DelW/PMMA. (H_2O was used in X-ray scattering).

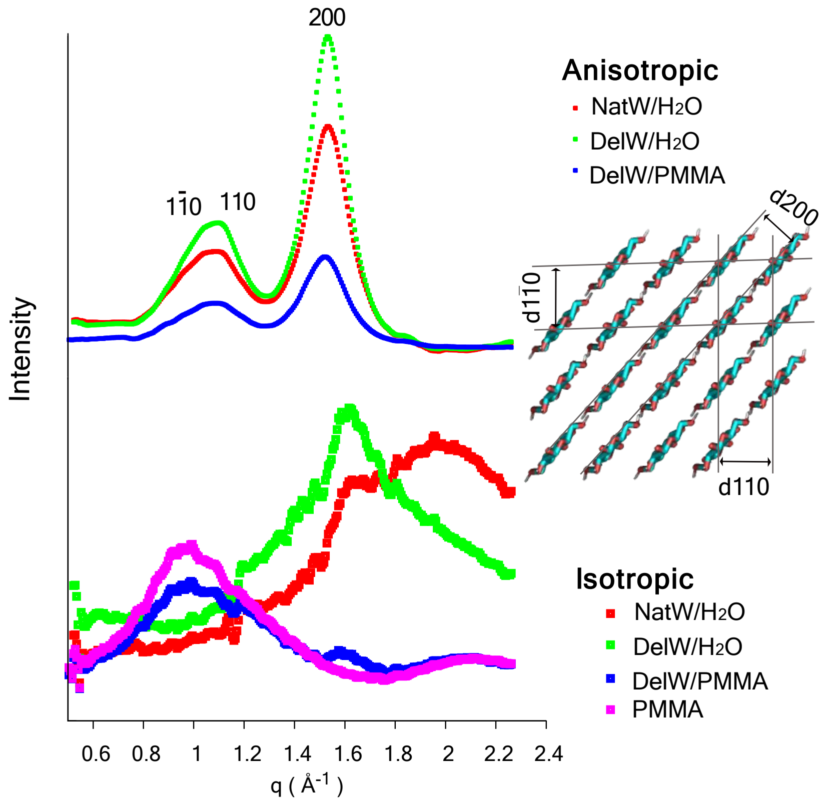

Figure S4: Decomposed WAXS profiles of NatW/H_2O, DelW/H_2O, DelW/PMMA and PMMA. (the decomposed profiles into anisotropic and isotropic scattering features show that transparent wood is composed of cellulose and PMMA, respectively; d1-10(0.6 nm), d110(0.54

nm), and d200(0.39 nm) refer to the three characteristic diffraction peaks of crystalline cellulose Iβ)

Part 2

Semi-quantitative interpretation of X-ray / neutron scattering peak position on woody samples

As the first approximation, we consider wood cell wall being constituted of uniform cellulose microfibrils with a radius *r* parallel packed in the cell wall embedded in a matrix of constant scattering length. Here we will discuss on the scattered intensity perpendicular to the microfibrils direction as a function of scattering vector *q*, the equatorial scattering $I_{eq}(q)$, which is simply proportional to the product of the contribution form factor of the fibril *F* and the structure factor *S* as

$$I_{eq}(q) = A|F(q)|^2|S(q)|^2 \qquad (1)$$

with *A* being an a scaling factor. If we approximate the microfibril as infinite cylinder, the form factor in this direction

$$F(q) = \mathrm{A}'\frac{J_1(qr)}{qr} \qquad (2)$$

Again with a proportionality factor *A'* for which we skip the detail, as we are not analyzing in absolute scale. J_1 is the 1st order Bessel function of the first kind.

$$Y = \left(\frac{J_1(qr)}{qr}\right)^2 \qquad (3)$$

is shown in Figure S5a, which shows an oscillation with a peak at around 5/r. *Y* represents the normalized form factor.

The structure factor depends on the arrangement of cylinder in the cell wall but since the cylinders are rigid, they cannot inter-penetrate. Thus the radial distribution function is 0 at distance up to two times the radius, and has a sharp peak at about this distance *2r*, followed by a second shell at slightly beyond *4r* and then converging to average number density, or unity at large distance (Fig. S5b). The Fourier transform of *g(r)* is the structure factor (Fig. S5c), now having a peak at around *π* and *2π*. The peak position of the structure factor is thus at the position far from the flat region of the form factor. This situation is very different from the scattering from densely packed atoms where lattice size are in general much larger, and thus the structure factor peak is much closer to the peak position of the atomic form factor where the form factor varies little as function of *q*.

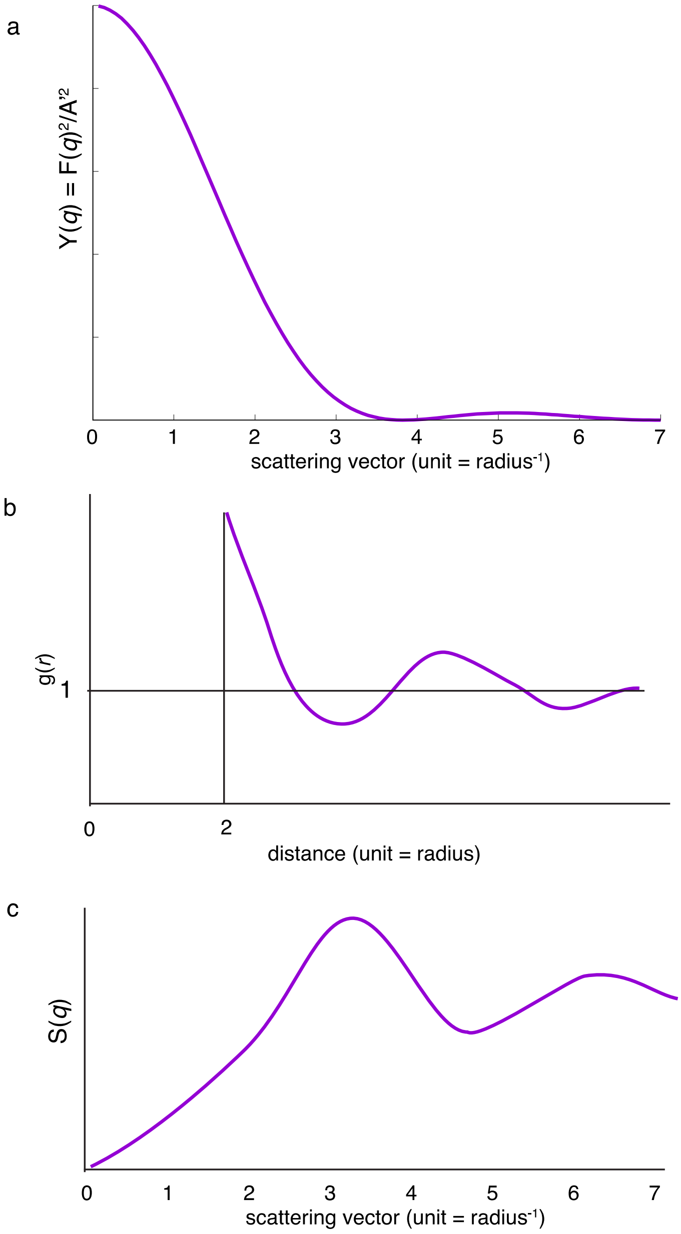

Figure S5. Schematic illustration of normalized (a)form factor, (b)pair distribution function, and (c)structure factor of densely packed cylinders.

The intensity we observe are the product of curve **a** and **c** in Fig. S5, and thus the first peak is actually observed at smaller q than the peak position of structure factor due to the descending slope of $|F(q)|^2$.

The effect of the slope of the form factor to the peak position can be estimated to the first approximation as parabolic function and straight segment. We place the peak at x=0 as

$$y = b - ax^2 \qquad (4)$$

, and see the effect of multiplying with ascending slope

$$y_1 = c - dx \qquad (5)$$

The position of the peak can be found from the derivative

$$\frac{d(yy_1)}{dx} = -d\left(b - ax^2\right) - 2ax(c - dx) \qquad (6)$$

$$= -3adx^2 - 2ax - bd = 0 \qquad (7)$$

and

$$x = \frac{-ac \pm \sqrt{(ac)^2 + 12\mathrm{ab}d^2}}{3\mathrm{ad}} \qquad (8)$$

We are interested at x ≈ 0, so

$$x = \frac{\mathrm{ac}\left(1 - \sqrt{1 + \frac{12abd^2}{(ac)^2}})\right)}{3\mathrm{ad}} \qquad (9)$$

$$\approx \frac{\mathrm{c}\left(1 - \left(1 + \frac{1}{2}\frac{12abd^2}{(ac)^2}\right)\right)}{3\mathrm{d}} \qquad (10)$$

$$= \frac{2bd}{ac} \qquad (11)$$

meaning that the shift is inversely proportional to the curvature (sharpness : *a*) of the structure factor peak and the form factor at peak position (*c*), and proportional the height of the structure factor (*b*) and the slope of the form factor (*d*). The net contribution of the form factor peak shift would be thus Y'/Y, that diverges when Y = 0 as shown in Fig. S6. At $q = \boldsymbol{\pi} / r$, the contribution of the form factor to the peak shift is significant.

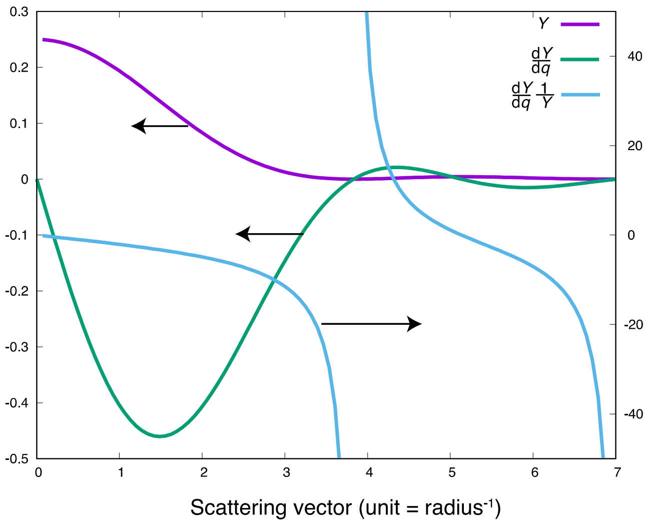

Figure S6. The effect of cylinder form factor on the peak position. Violet line is the cylinder form factor, green line is the derivative of form factor, blue line shows the contribution to the peak shift from the structure factor peak.

The sharpness of the structure factor peak depends on the extent of oscillation of the pair distribution function, which is expected to be attenuated faster when the packing density is lower. Thus, there is no analytical solution directly relating the observed peak position and the structure factor of the fibril arrangement and further analysis requires explicit model building.

Origin of the differences in peak position between X-ray and neutron scattering

In the above discussion we considered the scattering length density boundary to coincide with the hard cylinder. At molecular level, there are however nuances depending on the probe. For X-ray, the electron density centers of carbon and oxygen atoms are a few angstrom away from the boundary. For cell wall immersed in heavy water, the hydroxyl groups of the outermost chain will be exchanged to deuterium leading to higher scattering length density, whereas in non-aqueous system, this outermost chain is hydrogenated and shares the scattering length density of the core. Thus the difference in peak position between X-ray and neutron for the three cases can be explained as follows:

(1) Fully deuterated transparent wood

X-ray: The matrix electron density is lower than cellulose core, and thus the light atoms at the surface of the cellulose is seen as matrix. The effective cylinder diameter is smaller than the hard cylinder shifting the X-ray peak to higher *q*.

Neutron: the scattering length density of the matrix is high and the hydrogen of the surface of microfibril is counted as part of cylinder. The effective radius corresponds roughly to the hard boundary.

(2) Delignified wood in (heavy) water

X-ray: The matrix electron density is lower than cellulose core and the outermost light atoms are counted as matrix.

Neutron: The scattering length density dominated by water molecules is much higher than the cellulose core, but the surface hydroxyl groups are deuterated giving closer scattering length density to the matrix forming a transient layer.

In both cases, the effective cylinder diameter is smaller than the hard cylinder shifting the correlation peak to higher q compared to the hypothetical hard cylinder model.

(3) Native wood in (heavy) water

X-ray: similar to delignified wood

Neutron: Water in the cell wall is considered to be about 30% of the dry weight (fiber saturation point). As it does not penetrate into crystalline microfibrils corresponding roughly to half the cell wall substance, the matrix component can contain 60% of water of its dry-weight, thus occupying 40-50% of the volume, resulting in average scattering length density close to deuterated cellulose. Thus the surface chain can be considered as matrix leading to smaller effective cylinder diameter and the peak shifts to the higher q more than for X-ray.

References:

(1) Heller, W. T.; Urban, V. S.; Lynn, G. W.; Weiss, K. L.; O'Neill, H. M.; Pingali, S. V.; Qian, S.; Littrell, K. C.; Melnichenko, Y. B.; Buchanan, M. V.; Selby, D. L.; Wignall, G. D.; Butler, P. D.; Myles, D. A. The Bio-SANS Instrument at the High Flux Isotope Reactor of Oak Ridge National Laboratory. *J. Appl. Crystallogr.* 2014, *47* (4), 1238–1246. https://doi.org/10.1107/S1600576714011285.

(2) Nishiyama, Y.; Langan, P.; O'Neill, H.; Pingali, S. V.; Harton, S. Structural Coarsening of Aspen Wood by Hydrothermal Pretreatment Monitored by Small- and Wide-Angle Scattering of X-Rays and Neutrons on Oriented Specimens. *Cellulose* 2014, *21* (2), 1015–1024. https://doi.org/10.1007/s10570-013-0069-2.

(3) Berglund, L. A.; Burgert, I. Bioinspired Wood Nanotechnology for Functional Materials. *Adv. Mater.* 2018, 1704285. https://doi.org/10.1002/adma.201704285.

(4) Li, Y.; Fu, Q.; Yu, S.; Yan, M.; Berglund, L. Optically Transparent Wood from a Nanoporous Cellulosic Template: Combining Functional and Structural Performance. *Biomacromolecules* 2016, *17* (4), 1358–1364. https://doi.org/10.1021/acs.biomac.6b00145.

(5) Daicho, K.; Saito, T.; Fujisawa, S.; Isogai, A. The Crystallinity of Nanocellulose: Dispersion-Induced Disordering of the Grain Boundary in Biologically Structured Cellulose. *ACS Appl. Nano Mater.* 2018, *1* (10), 5774–5785. https://doi.org/10.1021/acsanm.8b01438.

(6) Daicho, K.; Kobayashi, K.; Fujisawa, S.; Saito, T. Crystallinity-Independent yet Modification-Dependent True Density of Nanocellulose. *Biomacromolecules* 2020, *21* (2), 939–945. https://doi.org/10.1021/acs.biomac.9b01584.

(7) Ehrnrooth, E. M. L. Change in Pulp Fibre Density With Acid-Chlorite Delignification. *J. Wood Chem. Technol.* 1984, *4* (1), 91–109. https://doi.org/10.1080/02773818408062285.
